# Mitigating antimicrobial resistance by innovative solutions in AI (MARISA): a modified James Lind Alliance analysis

**DOI:** 10.1038/s44259-025-00150-y

**Published:** 2025-09-01

**Authors:** William J. Waldock, Hannah Thould, Leonid Chindelevitch, Nicholas J. Croucher, César de la Fuente, James J. Collins, Hutan Ashrafian, Ara Darzi

**Affiliations:** 1 Institute of Global Health Innovation, Imperial College London, London, UK; 2 MRC Centre for Global Infectious Disease Analysis, School of Public Health, Imperial College London, London, UK; 3 Machine Biology Group, Institute for Biomedical Informatics, Institute for Translational Medicine and Therapeutics, Perelman School of Medicine, University of Pennsylvania, Philadelphia, PA USA; 4 Department of Biological Engineering, Massachusetts Institute of Technology, Cambridge, MA USA; 5 Broad Institute of MIT and Harvard, Cambridge, MA USA; 6 Wyss Institute, Harvard, Boston, MA USA

**Keywords:** Biological techniques, Computational biology and bioinformatics, Drug discovery, Environmental sciences, Diseases, Molecular medicine, Agriculture

## Abstract

Antimicrobial resistance (AMR) is a critical global health threat and artificial intelligence (AI) presents new opportunities for our response. However, research priorities at the AI-AMR intersection remain undefined. This study aimed to identify and prioritise key areas for future investigation. Using a modified James Lind Alliance approach, we conducted semi-structured interviews with eight experts in AI and AMR between February and June 2024. Analysis of 338 coded responses revealed 44 distinct themes. Major barriers included fragmented data access, integration challenges and economic disincentives. The top ten priorities identified were: Combination Therapy, Novel Therapeutics, Data Acquisition, AMR Public Health Policy, Prioritisation, Economic Resource Allocation, Diagnostics, Modelling Microbial Evolution, AMR Prediction and Surveillance. A notable limitation was the underrepresentation of data from high-burden regions, limiting the generalisability of findings. To address these gaps, we propose the novel BARDI framework: Brokered Data-sharing, AI-driven Modelling, Rapid Diagnostics, Drug Discovery and Integrated Economic Prevention.

## Introduction

Antimicrobial resistance (AMR) poses a grave and escalating threat to global health, with the potential to undermine the foundations of modern medicine, including chemotherapy, surgery and infection management^1^. Since 1990, AMR has been responsible for approximately one million deaths annually^2^, and projections from the Global Research on Antimicrobial Resistance (GRAM) Project^3^ estimate up to 1.91 million direct deaths per year by 2050. Overall, up to 39 million fatalities could be attributable to AMR by mid-century. The World Health Organization (WHO) has identified AMR as one of the top ten global public health threats^4^, warning that, without effective intervention, the economic toll could reach a staggering $100 trillion by 2050^5^.

Rising antibiotic consumption, expected to increase by over 30% globally by 2030^6^, exacerbates the crisis. The World Bank projects that AMR could cost the global economy between $1 and $3.4 trillion annually by 2030 and reduce global GDP by up to 3.8% annually by 2050^7^, pushing an estimated 28 million people into extreme poverty. The consequences are already being felt in the form of longer hospital stays, costlier treatments and increased mortality. In response to these alarming trends, global organisations have initiated urgent action. In 2019, AMR was formally recognised by the WHO as a threat to achieving Sustainable Development Goals^8^, and in 2023, a comprehensive global research agenda on AMR in human health was launched, outlining forty research priorities across prevention, diagnosis, treatment, care, policy and education^9^. Most recently, in September 2024, the United Nations General Assembly committed to reducing AMR-related deaths by 10% by 2030^6^, an ambitious target, especially given the considerable uncertainty in current estimates.

Amid these efforts, there is growing recognition that advanced technologies, particularly artificial intelligence (AI), could play a critical role in addressing AMR. From enhancing diagnostic accuracy and antimicrobial stewardship to optimising surveillance and accelerating drug discovery, AI offers promising tools in the global fight against resistance. AI is being rapidly applied across a broad spectrum of healthcare, from AI-augmented clinical research to algorithms for image analysis or disease prediction^4^. However, despite this surge in popularity, there has been no expert consensus or priority-setting exercise about the best applications of AI to AMR, nor a comprehensive examination of the barriers that currently prevent its implementation. This means there is the potential for an imbalanced focus of research, which may miss crucial areas for innovation and result in the perpetuation or creation of inequalities in healthcare. Work has been started on this through a Google DeepMind and Fleming Initiative publication ‘Harnessing Artificial Intelligence to Tackle Antimicrobial Resistance’^10^, in which the authors delineate six strategic pillars for leveraging AI to combat AMR: Priority Setting, Collaboration, Data, Evaluation, Capacity and Equity.

The absence of any formal priority-setting framework means current AI research into AMR is fragmented and risks missing crucial areas for potential research. Through this study, we generated a list of the top ten areas for potential research, identified barriers to research in applying AI to AMR and developed a framework for ongoing research. The primary aim of this study was to determine the areas where AI should be used in AMR research. To achieve this, the following objectives were established:

1. Determine the areas where AI could be used in AMR research.
2. Gain expert opinion on a list of priorities for AI in tackling AMR.
3. Determine the barriers that need to be overcome to apply AI to the problem of AMR.
4. Develop a framework for AI to be applied to reduce the problem of AMR.

## Results

**Fig. 1:**
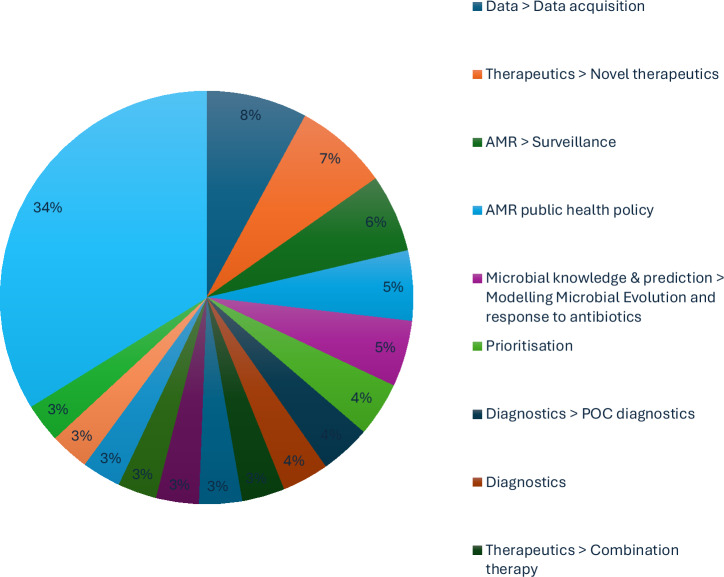
Thematic theme frequencies. See Supplementary Material.

A total of eight expert interviews were conducted, resulting in 338 coded responses and the identification of 44 unique themes. The most frequently referenced themes (Fig. 1) included:

1. Therapeutics—Combination Therapy
2. Therapeutics—Novel Drug Development
3. Data—Data Acquisition
4. AMR Public Health Policy
5. Research Prioritisation
6. Economic Resource Allocation
7. Diagnostics—Point-of-Care (POC)
8. Modelling Microbial Evolution
9. Microbial Knowledge and Prediction
10. AMR Surveillance

Table 1 presents illustrative quotes for selected subthemes. Most insights emphasised how AI can support AMR efforts across several domains: data challenges were the most prominent barriers, particularly regarding access, interoperability and data-sharing across institutions and borders; economic constraints were cited as limiting both innovation and implementation of AI-driven solutions; experts emphasised therapeutics, surveillance and point-of-care (POC) diagnostics as the most promising areas for AI deployment. Some participants reflected on lessons from the COVID-19 pandemic, particularly how AI-enabled tools accelerated diagnostics and monitoring, highlighting opportunities to replicate such successes in AMR. Thematic clusters revealed that experts often framed challenges in interconnected areas (e.g. economic modelling and microbial prediction), rather than isolating single issues. Precision medicine was frequently discussed in the context of high-risk populations (e.g. immunocompromised patients) and emerging interventions like phage therapy. Specific concerns were raised regarding lower- and middle-income countries (LMICs), where the lack of robust data and diagnostics may limit the global applicability of machine-learning models. Experts warned that data underrepresentation risks reinforcing inequities and undermining AI’s value in high-burden settings. Further research was also recommended at the microbial level, especially concerning the genome–phenome relationship under antimicrobial stress, to improve predictive modelling and diagnostics.

**Table 1:** Illustrative quotes

Subtheme	Illustrative quote
Data gaps	(1) The prediction and generation tasks we’ve outlined are data hungry… where are there mechanisms to gather quality training data?
Dataset coordination/data-sharing	(1) We need training datasets that merge chemical structure with biological and clinical outcomes, AI can’t learn robustly without that.
Data integration issues	(4) Public datasets are heterogeneous—ML models struggle because data from different labs aren’t standardised.
Conveying uncertainty	(3) ML models often give confident answers without conveying uncertainty… we need systems that know when they don’t know.
Possible solutions	(5) There are companies trying federated AI approaches… letting pharma share data without revealing proprietary info.
High-throughput screening	(4) With computers, we can now discover hundreds of thousands of antibiotic candidates in hours.
Combination therapies	(1) AI for predicting synergistic chemical combinations is ideal—but we lack the training data to do it reliably.
Drug repurposing	(1) If we train AI models across pharmacological properties, we can reuse what we’ve already done in other therapeutic areas.
Host factors	(7) AI could help predict which antibiotics will work across diverse human genotypes—but we need much better host-response data.
Vaccines	(3) Using smart statistical models to design vaccines is part of how AI could help us target high-risk resistant bugs.

### Application of the BARDI framework

The BARDI framework (Brokered data-sharing, AI-driven modelling, Rapid diagnostics, Drug discovery and Integrated economic prevention) was developed inductively through grounded thematic analysis of expert interviews. It offers a holistic, systems-level response to the complex challenges of applying AI to AMR. Rather than building on a pre-existing theory, the framework emerged directly from the data, with categories iteratively refined and overlapping themes merged (e.g. surveillance and data, or public communication and education).

### Brokered data-sharing

Experts consistently highlighted the need for secure, structured and scalable mechanisms to share datasets across sectors while protecting proprietary interests. A recurring concern was the fragmentation of data and the reluctance of pharmaceutical firms to share valuable screening information: ‘We need training datasets that merge chemical structure with biological and clinical outcomes… often the chemical space explored doesn’t overlap, so models can’t learn anything robust’. *(Expert 1);* ‘There are companies that do federated data sharing, acting as brokers between pharma groups. But in AMR, the incentives still aren’t there’. *(Expert 5)*. This underscores the critical role of brokered data-sharing as a foundational infrastructure for effective AI deployment.

### AI-driven modelling

Experts identified AI’s strengths in enhancing predictive modelling, from PK/PD predictions to global resistance forecasting and combination therapy design. However, the effectiveness of these models was repeatedly linked to data quality and integration: ‘ML models often give confident answers without conveying uncertainty… methods that admit when they don’t know are essential when removing phenotypic testing’. *(Expert 3);* ‘The application of AI for combination solutions is perfect. The issue is a lack of training data to predict synergistic interactions reliably’. *(Expert 1);* ‘We could train models across therapeutic areas; AI can reuse what we’ve already learned about PK/PD, solubility, etc., from oncology and other drug classes’. *(Expert 1)*. AI is thus positioned not as a plug-and-play solution but as a modelling engine that depends on cross-domain integration and robust uncertainty estimation.

### Rapid diagnostics

Rapid diagnostics were seen as a high-impact domain for AI, particularly in translating genomic data into actionable clinical decisions and enabling POC deployment in low-resource settings: ‘If you could sequence the pathogen genome and run it through CARD to predict susceptibility profiles… that’s the future of diagnostics’. *(Expert 7);* ‘The inference from genome data can be fast, but we need to move away from culturing to sequencing directly from messy samples which is where AI can help’. *(Expert 3);* ‘POC assays that not only identify the bug but say what you can treat with, those are urgently needed in LMICs’. *(Expert 8)*. These insights reinforce the role of AI in accelerating diagnostic inference, improving treatment precision, and supporting real-time surveillance integration.

### Drug discovery

Participants described a shift in antibiotic R&D, with AI facilitating high-throughput screening, small molecule prediction and vaccine design. However, translating in silico predictions into viable candidates remained a bottleneck: ‘Computers can now identify hundreds of thousands of preclinical candidates in hours, AI has compressed the early discovery timeline’. *(Expert 4);* ‘AI could help design molecules with antimicrobial activity and good pharmacological properties to basically do most of preclinical discovery computationally’. *(Expert 1);* ‘AI’s challenge is the human interface, you don’t just need to kill bacteria; drugs must be safe in humans at high concentrations’. *(Expert 7)*. Here, AI contributes by expanding the exploratory search space, enabling smarter triage of leads and optimising early-phase candidate pipelines.

### Integrated economic prevention

Finally, participants emphasised that AI and technological advances alone cannot resolve AMR without economic and behavioural infrastructure. Experts linked AI to resource targeting, risk stratification and evidence generation for stewardship policies: ‘If you’re an investor and someone pitches an antibiotic, you run. We need to treat AMR like a public good and use AI to show the payoff’. *(Expert 1);* ‘Precision medicine through AI can identify high-risk individuals, who should get prophylactic therapeutics for example’. *(Expert 6);* ‘Public awareness is still missing. AMR doesn’t have the narrative strength of cancer or ALS, we need storytelling, and AI can support that’. *(Expert 1)*. AI here enables economic prioritisation and public communication through improved modelling, population stratification and engagement strategies.

## Discussion

These results have set out the direction of expert opinion for AMR policy, particularly in how to optimise research and development efforts. Nevertheless, there remain obstacles to explore in implementing this expert vision.

**B**rokered data-sharing was proposed by the expert interviews to unify the divided efforts against AMR. A data broker is a regulator and diplomat; a referee of how data is collected and used, but also an unbiased arbitrator between commercially competitive organisations. This precedent is established in other industrial contexts by the UK Information Commissioner’s Office^11^ and European Data Protection Regulation^12^. Data was the most consistently raised issue throughout the interviews. The interviewees were aware that any machine-learning algorithm often requires large amounts of data, with concerns centred around access to datasets that already exist, particularly pharmacological data rather than microbial data. Possible solutions include a federated pharmacological dataset that does not compromise participating commercial research programmes, such as through a data broker. This would require standardisation of data collection or at least a data cleaning methodology. Moreover, Wan et al. demonstrated that AI models can be trained with less data than previously thought; APEX was trained on standardised experimental data collected for about 1000 compounds tested against an array of target bacteria, yielding a matrix of about 15,000 data points^13^.

**A**I-driven modelling is essential for the development of novel therapeutic options. Creating novel therapeutics was seen as a very high priority by interviewees, though most mentioned the importance of pursuing multiple avenues, as there is significant uncertainty as to where the future lies. Safety must be a central theme and concern. Alongside specifically targeting drugs, modelling pharmacokinetics and pharmacodynamics could also be approached using AI methods. Peptides, immunologics and small molecules were all seen as reasonable targets^14–16^. The paucity of anti-fungal therapy was highlighted as a pressing issue. Phage was viewed favourably in terms of a second-line therapy or personalised medicine, but concerns were raised about their longevity as a therapeutic option and the potential for unintended consequences (e.g. acting as a vector for AMR spread).

**R**apid diagnostics emerged as a key pillar in the fight against AMR. Interviewees emphasised the need for more accessible, affordable and rapid diagnostic tools that could be implemented at various levels of healthcare, including in low-resource settings. Advances in AI and molecular diagnostics can help enable real-time pathogen detection, reducing delays in treatment and limiting unnecessary antimicrobial use. Improved diagnostics would enhance antimicrobial stewardship, enable more precise epidemiological tracking and improve patient outcomes by ensuring the right treatment is administered early. Acquisition of this data needs to be standardised to optimise international efforts, which was also a subject of intense scrutiny during the AMR Data Symposium at The London School of Hygiene and Tropical Medicine in October 2024^17^. Data is currently stored in silos corresponding to traditional disciplines, and therefore, the datasets are incomplete, whilst there remain integration issues due to heterogeneity of data collection methods from different labs or disciplines. This is corroborated by a review of antimicrobial learning systems (ALSs), which noted the obstacles of incomplete data capture, dwindling pipelines of new antimicrobials, one-size-fits-all antimicrobial treatment formularies, resource pressures and poorly implemented diagnostic innovations^18^. Moreover, gaps in the currently available data require further research (e.g. pharmacological studies in humans), and data is being missed due to poor testing capability, resulting in skewed datasets. The specific priority for applying genomic data in the fight against AMR was also highlighted by the Wellcome conference on AMR in March 2024^19^. Comprehensive datasets are needed to fully realise the opportunities AI presents in combating AMR^20^.

**D**rug discovery is essential to deliver regional biosecurity. Vaccines were highly rated by interviewees, but they acknowledged the difficulty of creating anti-bacterial rather than anti-viral vaccines. Nevertheless, it is appealing to target organisms that drive high antimicrobial use. Therapeutic approaches were an area most interviewees felt was a priority, though it tended to be highlighted by those who were currently working in this field, and they admitted their bias due to their specialisation. The concept of automating preclinical drug discovery was seen as very appealing. High-throughput screening was detailed as a way that AI could be used to speed up screening of vaccine candidates and to highlight new potential mechanisms for drug targets. Combination therapies were described as a way that AI was well-suited to the large-scale screening required for finding combination therapies. However, the challenges raised by interviewees, which mostly centred around pharmacokinetic and pharmacodynamic concerns, should be noted. However, although a drug may work synergistically with another in theory, the concentrations required for antimicrobial drugs, rather than other non-antimicrobials, are often greater by an order of magnitude, which presents safety concerns. AI was postulated to model the factors that affect drug penetration, the effect of the immune system and the effect of the gut microbiome.

**I**ntegrated Economic Prevention refers to the strategic investment in preventative public health measures that mitigate financial and societal costs associated with AMR before they escalate into more severe economic and healthcare burdens. It underscores the importance of cost-effective interventions, such as improved stewardship, surveillance and public awareness campaigns, that not only reduce long-term expenditures for healthcare systems but also enhance societal engagement by demonstrating tangible financial savings. Economic prevention is an essential component of framing the public narrative. The cost of AMR and the savings to be made for the public purse through pre-emptive action are needed for improving societal engagement. Despite the ongoing research and predicted professional concern, AMR does not have the public profile of other diseases, such as cancer.

This BARDI framework for directing AMR policy is corroborated by leading reviews in this space. To that end, Rabaan et al.^21^ discuss how AI can address AMR by optimising antibiotic prescriptions, predicting resistance patterns and enhancing diagnostics; they emphasise the potential of machine learning to improve clinical decisions and reduce the misuse of antibiotics. Moreover, Howard et al.^18^ propose a framework for implementing AI-driven ALSs, and highlight AI’s role in optimising antimicrobial use, addressing the complexity of AMR data and promoting adaptive, sustainable interventions within healthcare systems; the development of ALSs is especially important for modelling in surveillance efforts and understanding the drivers of AMR^22^. Meanwhile, Ali et al.^23^ outline AI’s potential in antimicrobial stewardship, emphasising its application in predicting bacterial resistance, optimising treatment pathways and identifying emerging threats through large-scale data analysis. Furthermore, Rabaan et al.^21^, in alignment with the BARDI framework, focus on AI’s application in clinical settings, particularly in improving antibiotic stewardship. Moreover, Ali et al.^23^ further support these views by detailing AI’s role in data-driven antimicrobial stewardship, reinforcing the importance of large-scale data integration and machine learning for proactive AMR management. Moreover, inappropriate antibiotic prescribing leads to increased AMR, patient morbidity and mortality. Clinical decision support systems that integrate AI with widely used clinical screaming systems can aid clinicians by providing accurate, evidence-based recommendations. This would enable more objective prescription of antibiotics in compliance with guidelines as per diagnosis. To this end, Howard et al.^18^ expand on this by proposing a systemic framework for integrating AI within healthcare, stressing the importance of continuous learning systems. The obstacles to progress in AI use for AMR include incomplete or biased data, especially from primary care, which hinders accurate AI predictions. Sparse data and fragmented health systems complicate AI’s effectiveness^18,20^; the BARDI framework provides a basis for international cooperation against AMR.

Moreover, this BARDI framework aligns with Chindelevitch et al.^24^ who describe AMR advancements in surveillance, prevention, diagnosis and treatment; this research group has developed the first WHO-endorsed catalogue of mutations in the *Mycobacterium tuberculosis* complex associated with drug resistance and also developed INGOT-DR, an interpretable machine-learning approach to predicting drug resistance in bacterial genomic datasets^25^. The molecular mechanisms of AMR have been reviewed to deliver an overview of intervention and modelling opportunities, particularly using genomic sequencing and functional genomic approaches, including CRISPR^26^. Machine-learning applications to whole-genome studies^27^ present opportunities for both AMR surveillance as well as downstream drug discovery. Whilst applying AI to small molecules may generate recommendations which are not chemically synthesizable^28^, future models may be able to incorporate pharmacology and toxicity to deliver promising molecules such as Guavanin-2, which expressed the novel and non-coded mechanism of hyperpolarization of the bacterial membrane^29^. Meanwhile, with a growing demand for microbial resistance data collection, the irregular standards and practices of reporting globally compromise confidence in the field’s advancement^30^. Modelling AMR transmission may enable personalised antimicrobial stewardship interventions, particularly in *carbapenem-resistant Klebsiella pneumoniae*^31^. This necessitates a whole-system approach^32^ with collaborations such as the Surveillance Partnership to Improve Data for Action on Antimicrobial Resistance [SPIDAAR], funded by Pfizer and the Wellcome Trust^33^, for national plans for upscaling surveillance and prevention programmes in collaborations with private sector resources, particularly for deriving mechanistic insights on drug activity from population-scale experimental data^34^.

This BARDI framework is an urgent strategy to fight AMR; in a study of 41.6 million US hospital admissions (over 20% of national hospitalisations annually) between 2012 to 2017, the incidence of extended-spectrum beta-lactamase increased by 53.3% (from 37.55 to 57.12 cases per 10,000 hospitalisations), demonstrating that most hospitalisations were now showing characteristics of AMR^10^. If this trajectory does not stop, even the wealthiest nations will be overwhelmed.

There are at least four ongoing considerations to the implementation of this BARDI framework. Data privacy and security are important when dealing with sensitive patient information. Global standardisation and interoperability of AI systems are necessary to ensure that AI-driven AMR detection can be effectively integrated for biosecurity across different healthcare environments^35^. Now that 460 academic, commercial and public data consumers in the UK National Health Service have been mapped, and the challenges of multistage data flow chains have been shown to include noncompliance with best practice, there remain concerns about delivering the full potential of UK health data^36^, including the establishment of a data broker.

Subtle differences in the definitions of a particular clinical event can have a dramatic impact on prediction performance. Cohen et al.^37^, when applying tree-based, deep learning and survival analysis to the MIMI-III intensive care admissions database, demonstrated a 0-6% variation in area under the receiver operator characteristic (AUROC) depending on the onset of time in different definitions of sepsis. This is an essential observation to consider when using AI in the forecast of AMR because of the potential for resistance to be expressed in different patterns across different bacteria, as each resistant strain manifests harm to the host. A more precise definition beyond ‘when microbes no longer respond to antimicrobial medicines’^38^ is needed to minimise the variation in AUROC of AMR predictive diagnostics; there must not be a repeat of the error of the Sepsis-III definition in not defining the clinical event onset time^39,40^. Moreover, it is important to differentiate suspicion of infection and organ dysfunction when interpreting the clinical scenario^41^; verifiable methods with a precise definition of AMR are needed to direct a valid clinical response. Li et al.^42^ may have begun to address this problem by cataloguing some of the exact alleles associated with specific resistance patterns, but this would need to be improved to real-time analysis to allow for meaningful improvements to clinical decision-making.

Both ‘One Health’ and ‘Global Health’ are interdisciplinary concepts based on the interdependence of human and animal health, and integrating biological, environmental and socioeconomic factors, but they address AMR at different levels. Hospitals, patients and infections are all sometimes described as ‘resistant’^43^, but technically only the bacteria become resistant to an antibiotic^38^. However, when interrogating genomic and EHR data to develop a forecast, one is inclined to define AMR by a broader criterion of observed phenomena as the infection takes effect in the host patient and hospital. Gene capture strategies^44^ and real-time PCR procedures^45^ may improve the detection of antibiotic-resistant bacteria, regardless of their environment. This matters for a ‘Global Health’ perspective since novel resistance arises in one place and then disseminates worldwide^46^.

Modern vaccine platforms such as mRNA, protein subunit and vector-based technologies offer new opportunities for developing targeted vaccines against resistant pathogens, alleviating the AMR pressure on healthcare systems worldwide^47^. Vaccines offer a complementary approach to antimicrobials by decreasing antibiotic demand; vaccines against *Streptococcus pneumoniae* and *Haemophilus influenzae type b* have reduced rates of pneumonia and meningitis^48^. This reduces demand for broad-spectrum antibiotics, slowing resistance from antibiotic regimens selecting for resistant strains^49^. Moreover, vaccination against viral pathogens can help reduce the incidence of secondary bacterial infections and reduce symptomatic presentations, which may be confused with bacterial pathology^50^. In populations with high influenza vaccination coverage, there is a consequent reduction in antibiotic prescriptions, particularly during flu seasons^51^. mRNA vaccines, vector-based vaccines and protein subunit vaccines have expanded the potential for targeting AMR directly^52^. mRNA vaccines use messenger RNA to induce cells to produce specific antigens, thereby triggering an immune response to AMR pathogens^53^. This technology allows for the rapid development and testing of vaccines against AMR bacteria with rapid turnover, such as *Pseudomonas aeruginosa* and *Mycobacterium tuberculosis*^54^. Protein subunit vaccines enable targeted responses against resistant bacterial strains, such as *MRSA*^55^. Conjugate vaccines, which combine a protein with a polysaccharide to enhance immunogenicity, can prevent infections that commonly exhibit antibiotic resistance; the pneumococcal conjugate vaccine has not only reduced pneumococcal disease prevalence but also curbed the spread of drug-resistant *S. pneumoniae* strains^56^. However, caution is required as some drug-resistant strains have evaded vaccination^57^ and general resistance has been reported to bounce back to pre-vaccine levels^58^. Vector-based vaccines can be engineered to target specific resistant pathogens, especially those with complex life cycles^59^. *Escherichia coli*, *Klebsiella pneumoniae* and *Acinetobacter baumannii* are emerging targets for pharmaceutical research^60^ since these infections are difficult to treat due to multidrug resistance.

Moreover, these developments complement combination machine-learning methodologies, which leverage data-driven models in synthetic gene circuit engineering^61^; the introduction of RhoFold+^62^, an RNA language model-based deep learning method that accurately predicts 3D structures of single-chain RNAs from sequences, enables the fully automated end-to-end pipeline for RNA 3D structure prediction. Vaccines could significantly impact AMR by preventing infections that are notoriously hard to manage with available antibiotics. Whilst we wait for vaccines to be developed, resistant microorganisms will need to be scrutinised for druggable vulnerabilities, such as through collateral sensitivity induction by manipulating bacterial physiology^63^. These intermediate efforts can be supported by antimicrobial resistant gene databases^64–69^, particularly which offer spatiotemporal and abundancy data^70^.

The full James Lind Alliance (JLA) protocol process was modified; each Priority-Setting Partnership (PSP) would normally consist of patients, carers and their representatives, and clinicians, and is led by a Steering Group. The Steering Group oversees the activities of the PSP and has responsibility for the activity and the outcomes of the PSP. Since the role of the PSP is to identify questions that have not been answered by research to date, this study followed a modified path with the experts giving answers within a thematic framework. While the study excluded direct public involvement in its priority setting, it acknowledges that public awareness and engagement are critical in combating AMR. Instead of broad public surveys and workshops, the study used targeted expert interviews to identify research priorities in applying AI to AMR. Despite this abridged process, the extensive subsequent thematic analysis maintained the principles set out by the National Institute for Health and Care Research, which coordinates the infrastructure of the JLA to oversee the processes for PSPs, based at the NIHR Coordinating Centre, University of Southampton.

The BARDI framework advances previous antimicrobial-resistance proposals by transforming disconnected objectives into a unified, action-oriented ecosystem. Whereas previous action plans simply urged stronger surveillance, more research, better diagnostics, expanded R&D and the development of an economic case for investment, BARDI weaves these goals into five interlocking pillars that actively drive progress. First, instead of leaving data systems in siloed national or sectoral hands, BARDI establishes a neutral data broker. This broker enforces common standards, mediates access and protects proprietary interests, ensuring that human health, veterinary, agricultural and environmental datasets flow together under fair governance. Second, BARDI elevates AI from an aspirational research tool to the central engine of the response: AI-driven modelling continuously ingests brokered data to forecast resistance trends, optimise pharmacokinetic/pharmacodynamic regimens and even propose novel therapeutic compounds. Third, rapid diagnostics are promoted from a side objective to a full pillar. Decentralised, POC tests feed real-time results into the data broker, triggering AI-informed stewardship interventions and improving surveillance with minimal delay. Fourth, the drug-discovery pillar couples AI-identified bacterial targets with a prioritisation schema linked directly to public health impact, ensuring that investment goes toward the most critical vaccines and antimicrobial compounds rather than dispersing funding across undifferentiated R&D. Finally, integrated economic prevention converts the longstanding call to ‘develop the economic case’ into a dynamic funding mechanism. Real-time data on resistance burden and projected economic losses guide the allocation of resources to stewardship programmes, awareness campaigns, incentive prizes and cross-sector collaborations. In this way, economics is not merely a justification for action but an operational tool that continually sustains and adapts the entire BARDI ecosystem.

The top ten expert research themes for the application of AI to AMR provide valuable clarity on allocating resources to inform research developments in biosecurity and pandemic preparation. We propose a BARDI framework for directing AMR policy (Brokered data-sharing, AI-driven modelling, Rapid diagnostics, Drug discovery and Integrated economic prevention). Data was the most consistently raised issue throughout the interviews and needs to be neutrally coordinated to avoid obstacles, including automation bias, fear of job displacement and scepticism about AI’s decision-making abilities. Developing AI for AMR faces complex challenges, particularly in ensuring that AI tools comply with medical device regulations and data protection laws across different countries. The BARDI framework is a coherent strategy to fight AMR with AI to help realise the aspiration of the GRAM Project to save up to 92 million lives by 2050^3^.

## Methods

This study employed a modified JLA approach between February and June 2024 to identify research priorities at the intersection of AI and AMR. Unlike traditional JLA PSPs, which involve patients, carers and clinicians, this study included only domain experts. This was due to the technical nature of AI applications to AMR diagnostics and interventions, which require specialist knowledge. Nevertheless, the importance of public engagement in AMR remains acknowledged, and complementary work is being conducted at the Institute of Global Health Innovation (IGHI) to address this gap. The COREQ^71^ (Consolidated Criteria for Reporting Qualitative Research) 32-Item Checklist is set out in the Supplementary Material.

### Study design and participant recruitment

Participants were purposively selected based on their recognised expertise in either AI or AMR. Recruitment was conducted via a professional academic email. Invitations included a synopsis of the study and instructions for participation. A short slide deck outlining possible AI applications to AMR was sent in advance to structure the interview conversation; this material is available in the Supplementary Material. Eligible participants included professionals with demonstrated experience in research or policy related to AMR and/or AI. Informed consent was obtained from all participants. All necessary approvals were granted by the Imperial College Research Ethics Committee (ICREC), with authorisation from the Head of Department. A total of 23 domain experts were invited to participate in this study. Of these, eight agreed to take part, yielding a response rate of 35%. Participants were purposively selected based on their recognised expertise in AMR, AI, microbiology, clinical practice and health systems research. All participants held senior academic or clinical positions and were actively engaged in research at the intersection of AI and infectious diseases.

Participants represented a range of disciplinary backgrounds, including AI and machine learning, bioinformatics, microbial genomics and evolution, molecular microbiology, translational medicine, pharmacology and global health. Thematic areas of expertise included AI for precision antimicrobial prescribing, the development and implementation of diagnostic and surveillance technologies, understanding the molecular mechanisms of resistance (e.g. efflux pumps, resistance gene regulation) and health systems approaches to antimicrobial stewardship. Although the majority of participants were based at institutions in high-income countries (notably the United Kingdom, the United States of America and Canada), several had significant experience working in or collaborating with research teams in LMICs, including leadership roles in global initiatives with WHO-affiliated programmes. This ensured that global equity considerations and contextual diversity were reflected in the priority-setting process.

A summary of participant characteristics and research areas is provided in Supplementary Table S1.

### Data collection

All interviews were conducted online, averaged 45–60 min, and were followed by asynchronous email input. We acknowledge that while thematic saturation was largely achieved, the relatively small sample (*n* = 8) represents a limitation. Interviews were semi-structured and conducted either online or by phone, depending on participant preference. Conversations were guided by predefined themes, including:

- Evidence gaps and uncertainties in AMR
- Antibiotic and drug design
- Novel/non-classical drug solutions (e.g. biologics, phage therapy)
- Combination therapies
- Biomarker and point-of-care (POC) test design
- Drug repurposing
- Precision and population-level prediction
- Behavioural modification
- AMR surveillance and epidemic prediction
- Economic resource allocation
- Health policy development
- Big data analytics and real-world evidence
- Communication of AMR challenges

Interviews were audio-recorded with permission and transcribed verbatim. Follow-up email exchanges were used to clarify and expand on responses, with these data included in the thematic analysis. Participants were asked a series of open-ended questions to elicit expert insight on the opportunities and limitations of AI in addressing AMR. Illustrative questions, as shown in the Supplementary Slide Deck and ‘Topic Guide for MARISA’, included: ‘What is your understanding of AI and its applications to AMR?’, ‘Are there areas within AMR where AI would be particularly amenable or, conversely, unhelpful?’ and ‘What barriers currently hinder the application of AI to AMR?’. Experts were also asked to reflect on a pre-identified list of thematic areas, such as prevention, diagnosis, treatment, surveillance and stewardship, and were prompted to indicate priorities, gaps, or overlooked domains (‘Do any stand out?’, ‘Are there areas we should prioritise?’, ‘Any areas that are not on this list?’). In addition, participants were invited to suggest enablers for progress, with prompts focused on policy goals, data infrastructure and education.

Responses were thematically coded using an inductive approach. Each transcript was first open-coded line-by-line, and emerging codes were then grouped into higher-level categories representing key domains of interest. For example, responses regarding AI’s usefulness in predicting antimicrobial susceptibility were coded under ‘diagnostic applications’, while suggestions to improve training and digital literacy were grouped under ‘capacity building and education’. Barriers such as data fragmentation, algorithmic bias, or regulatory uncertainty were captured under ‘infrastructure and governance’ and ‘ethical and legal challenges’. Codes were iteratively refined and clustered into broader categories to inform the final prioritisation framework.

### Data analysis

An inductive thematic analysis approach was employed to analyse the qualitative data, facilitated by NVivo software. This method was chosen to allow themes to emerge from the data without imposing pre-existing categories, ensuring that the analysis remained grounded in participant perspectives. Two researchers (W.W. and H.T.) independently coded all interview transcripts using an open coding approach. After initial coding of the first few transcripts, the researchers held consensus meetings to compare and discuss their interpretations of emerging codes. These discussions informed the iterative refinement of a shared coding framework, which was then applied to the full dataset. To ensure reliability, selected transcripts were double-coded, and discrepancies in code application were systematically discussed and resolved during regular reviewer meetings. This process helped to clarify code boundaries and improve consistency in thematic categorisation.

### Justification for expert-only focus

This study focused exclusively on experts due to the complex and technical nature of AI’s application to AMR, a field in which public input, while valuable, may not sufficiently inform priority-setting at the technical level. That said, broader public engagement remains a parallel focus of the IGHI’s ongoing AMR work. Unlike the usual JLA PSPs, which involve patients, carers and clinicians, this study only included experts in AMR and AI. This decision was made because the application of AI to AMR diagnostics is a technical issue requiring specialist knowledge rather than direct public involvement. While the study excluded direct public involvement in its priority setting, it acknowledges that public awareness and engagement are critical in combating AMR. Instead of broad public surveys and workshops, the study used targeted expert interviews to identify research priorities in applying AI to AMR. Public involvement is so important that separate work from the IGHI is working specifically on how to engage the public on AMR issues; the goal is to improve awareness of AMR as a global health threat, influence behavioural changes in antibiotic use and ensure that funding and policies align with both expert recommendations and public concerns.

The inclusion criteria were professionals with expert knowledge of AMR and/or AI, as identified by the study team, who gave explicit consent to participate. The Principal Investigator obtained approval from the Head of Department and a favourable opinion from ICREC.

## Supplementary information

Supplementary Information

## Data Availability

Data are provided within the manuscript or Supplementary Information files.
