# Supplementary material for: Mitigating antimicrobial resistance by innovative solutions in AI (MARISA): a modified James Lind Alliance analysis
Source: NPJ Antimicrob Resist. 2025 Sep 1;3:75. doi: 10.1038/s44259-025-00150-y (PMC12402447; doi:10.1038/s44259-025-00150-y)
Supplement: Supplementary file 1 — Supplementary Information [file 44259_2025_150_MOESM1_ESM.pdf]

INSTITUTE OF GLOBAL HEALTH INNOVATION

Imperial College London

Mitigating Anti-microbial Resistance by Innovative Solutions in AI (MARISA)

Hutan Ashrafian
Lead for AI and Big Data, IGHI
hutan@ic.ac.uk

INSTITUTE OF GLOBAL HEALTH INNOVATION

Global Risk

Imperial College London

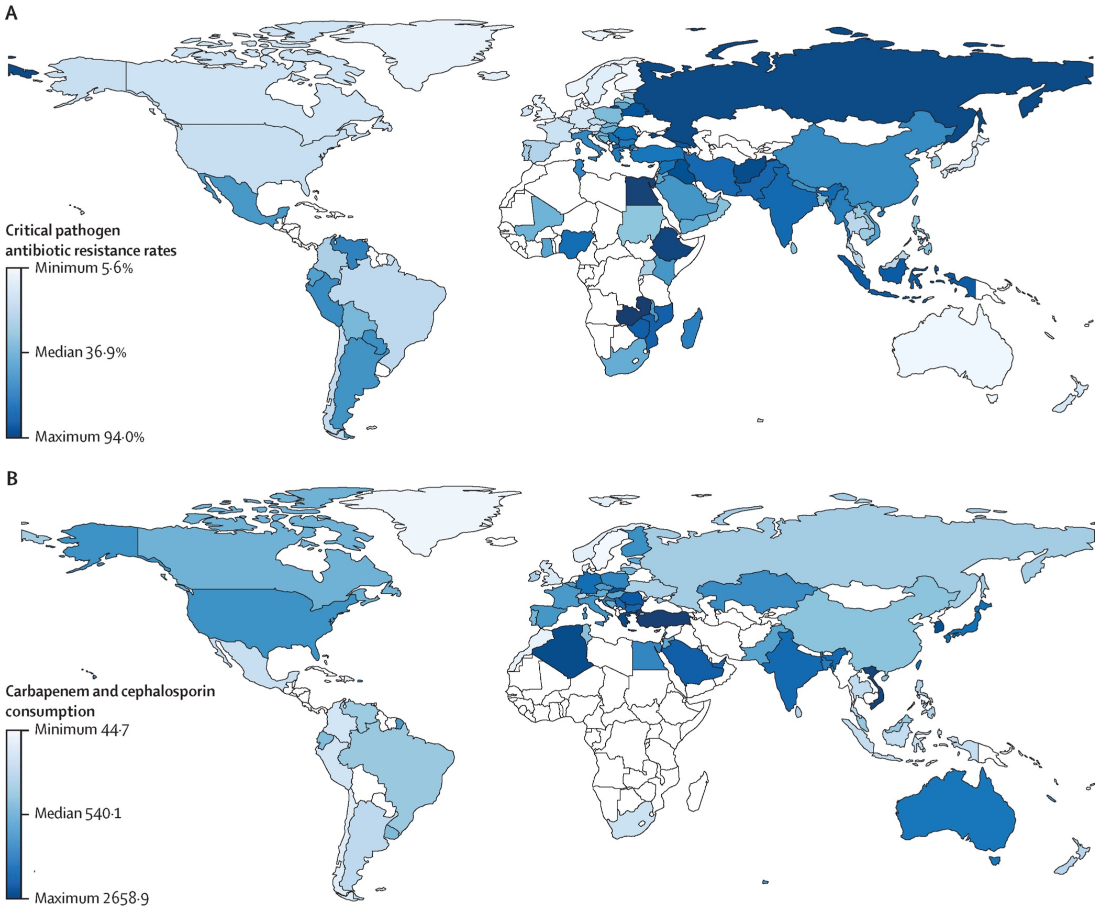

INSTITUTE OF GLOBAL HEALTH INNOVATION

Consensus Priority Settings

Imperial College London

Phase I

- Objective: Gather uncertainties (questions insufficiently addressed by current research)
- Method: Survey World Experts and identify supporting literature

Phase II

- Objective: Develop a short list of uncertainties
- Method: Interim prioritization survey of stakeholders

Phase III

- Objective: Arrive at a top list of research priorities
- Method: Remote/Email prioritisation process

Evidence Gaps and Uncertainties in AMR

INSTITUTE OF GLOBAL HEALTH INNOVATION

Imperial College London

(i)Antibiotic/Drug Design

(ii)Novel/non-classical-drug solutions, eg biologics and other technologies

(iii)Combination solutions

(iv)Biomarker design (population)

(v)POC tests

(vi)Antibiotic/Drug re-purposing

(vii)Precision medicine/individual prediction

(viii)Population prediction/identification of high-risk individuals (genomics/EHR)

(ix)Behavioural modification

(x)AMR Surveillance/epidemic surveillance

(xi)Economic Resource allocation

(xii)Health policy development

(xiii)Big Data Analytics and Real World Evidence

(xiv)Communication of AMR

TOPIC GUIDE FOR MARISA 2:

From MARISA:

Evidence Gaps and Uncertainties in AMR:

i. Antibiotic/Drug Design
ii. Novel/non-classical-drug solutions, eg biologics and other technologies
iii. Combination solutions
iv. Biomarker design (population)
v. POC tests
vi. Antibiotic/Drug re-purposing
vii. Precision medicine/individual prediction
viii. Population prediction/identification of high-risk individuals (genomics/EHR)
ix. Behavioural modification
x. AMR Surveillance/epidemic surveillance
xi. Economic Resource allocation
xii. Health policy development
xiii. Big Data Analytics and Real World Evidence
xiv. Communication of AMR

Also:

- Clinical uses and targets
- Antimicrobial materials and engineering
- Preventative medicine?

Need to state:

- Interviewee title:
- Area of expertise:
- Seniority:
- Institution:
- Gender:

Questions:

- What is your understanding of AI and its applications to AMR?
- This is a list of areas we could target:
    - Do any stand out?
    - Are there any areas that we should prioritise in terms of importance?
    - Are there areas which would be particularly amenable to AI?
    - Are there areas where AI would not be helpful?
- Are there any barriers to the application of AI to AMR?
- What do you think we need to put in place in order to be able to progress with AI research in this area?
    - Policy goals
    - Data
    - Education
- Any areas that are not on this list?

Supplementary Table S1. Participant Characteristics and Thematic Expertise

Characteristic	Details
**Number invited**	23
**Number participated**	8
**Response rate**	35%
**Geographic focus**	Primarily HICs (UK, US, Canada); global partnerships including LMICs
**Disciplinary backgrounds**	AI/ML, bioinformatics, microbiology, pharmacology, genomics, medicine
**Institutional affiliations**	University of Oxford, Harvard, Stanford, Cambridge, McMaster, UCL, others
**Key thematic areas**	
– AI and precision prescribing	
– AMR surveillance and pathogen genomics	
– Antibiotic resistance mechanisms	
– Antimicrobial stewardship	
– Translational medicine and vaccines	
– Computational biology and informatics	
– Health systems and global policy	

COREQ (Consolidated Criteria for Reporting Qualitative Research) 32-Item Checklist

Domain 1: Research Team and Reflexivity

1. Interviewer/facilitator: HT conducted the interviews.
2. Credentials: WW and HT are experienced researchers in qualitative methods and health data science.
3. Occupation: Both were PhD students in AI and AMR at Imperial College London.
4. Gender: HT identified as female and WW as male.
5. Experience and training: Both received formal training in qualitative research methods and had prior experience conducting interviews.
6. Relationship established: No prior relationships existed with participants.
7. Participant knowledge of the interviewer: Participants were informed of the researchers' roles and study aims through the invitation email and slide deck.
8. Interviewer characteristics: Both interviewers had academic backgrounds and no conflicts of interest with participants.

Domain 2: Study Design

9. Methodological orientation and theory: Inductive thematic analysis.
10. Sampling: Purposive sampling based on expertise in AMR or AI.
11. Method of approach: Via professional academic email.
12. Sample size: Eight experts participated.
13. Non-participation: Fifteen invited did not participate.
14. Setting of data collection: Online or by phone, based on participant preference.
15. Presence of non-participants: No.
16. Description of sample: Experts in AI or AMR, with diverse subfields including therapeutics, policy, and diagnostics.
17. Interview guide: Yes, a semi-structured guide and slide deck were used.
18. Repeat interviews: No.
19. Audio/visual recording: Yes, audio recording was used.
20. Field notes: Yes, supplementary notes were taken.
21. Duration: Interviews varied in length but typically lasted 30–45 minutes.
22. Data saturation: Saturation formally declared.
23. Transcripts returned: No.

Domain 3: Analysis and Findings

24. Number of data coders: Two (WW and HT).

25. Description of the coding tree: Not included in the manuscript but available upon request.

26. Derivation of themes: Themes were derived inductively from the data.

27. Software: NVivo.

28. Participant checking: No.

29. Quotations presented: Yes, see Table 1.

30. Data and findings consistent: Yes.

31. Clarity of major themes: Yes.

32. Clarity of minor themes: Yes, but quotes were summarised.
